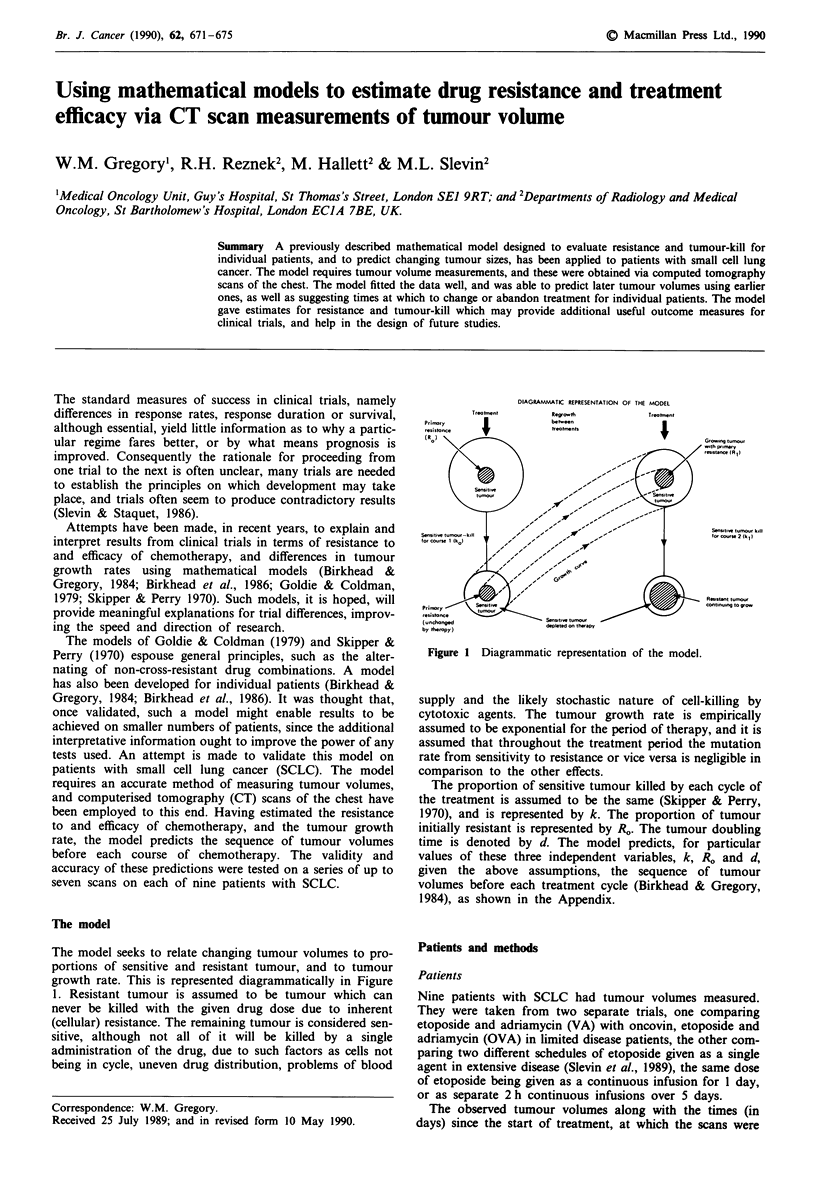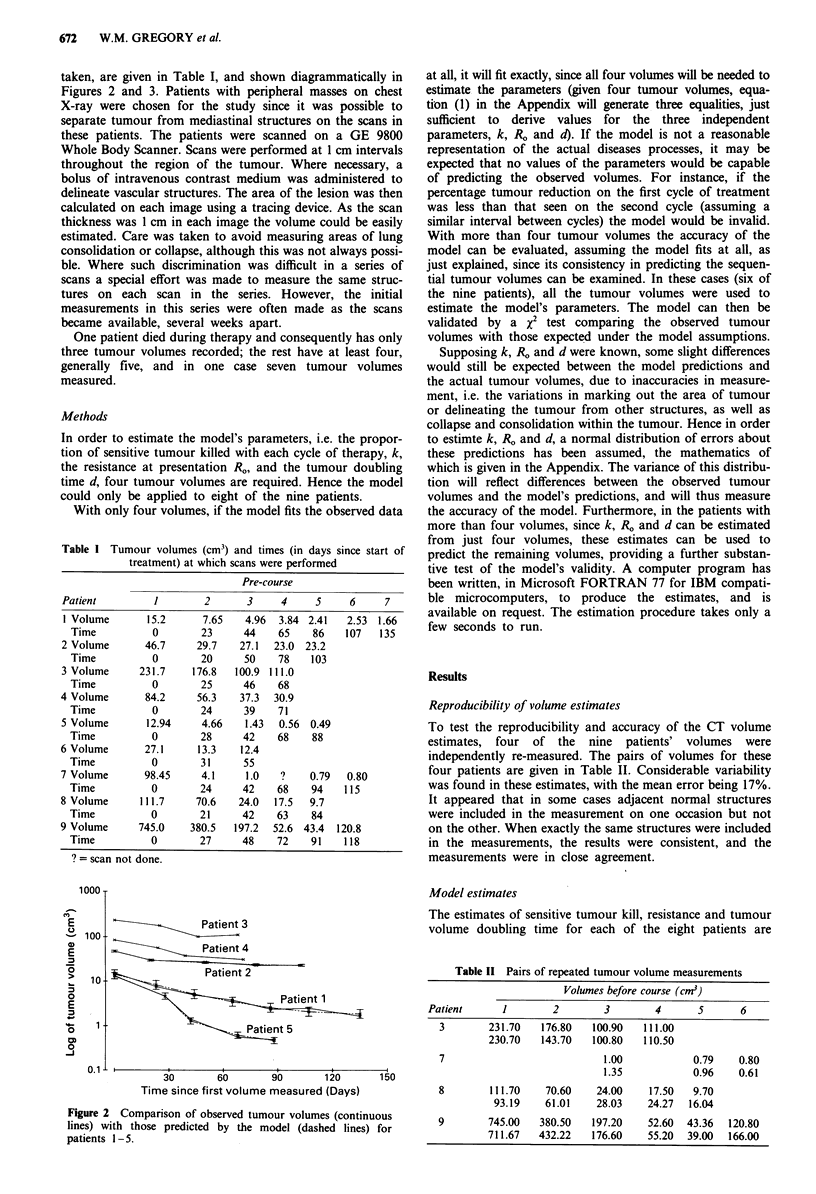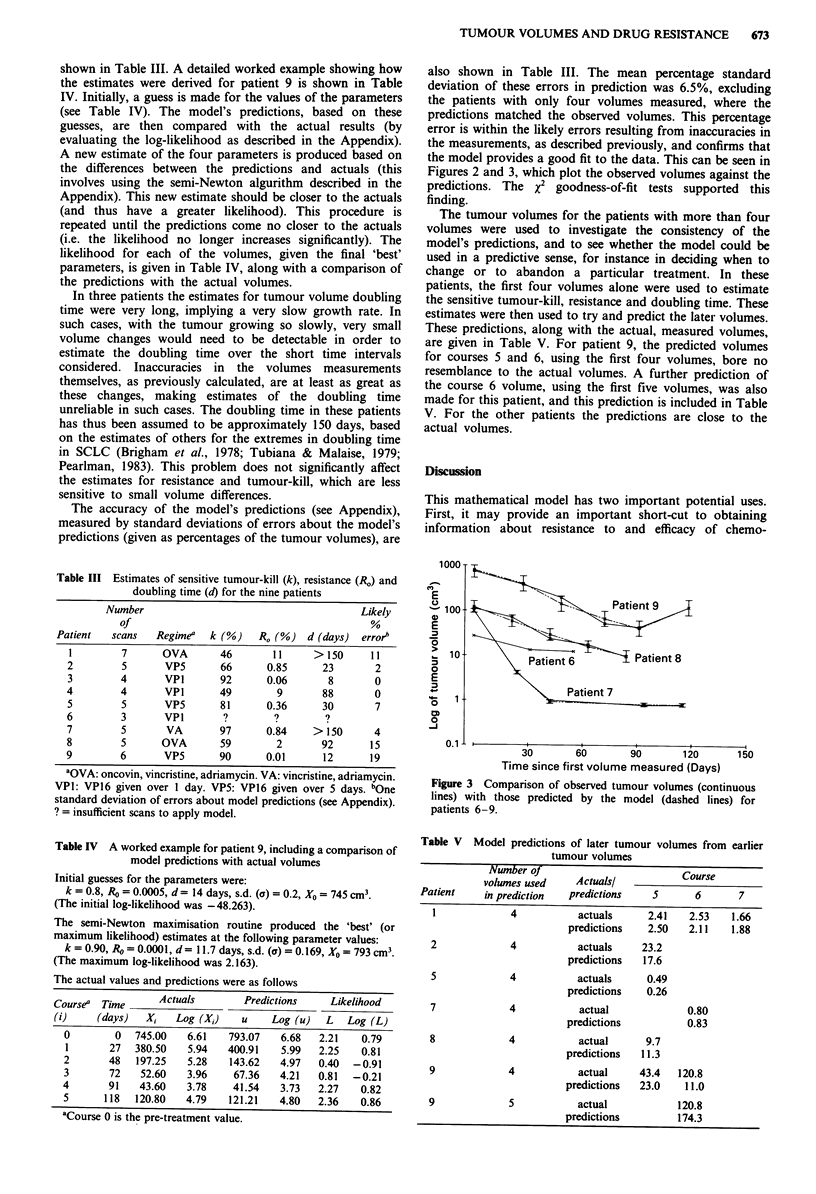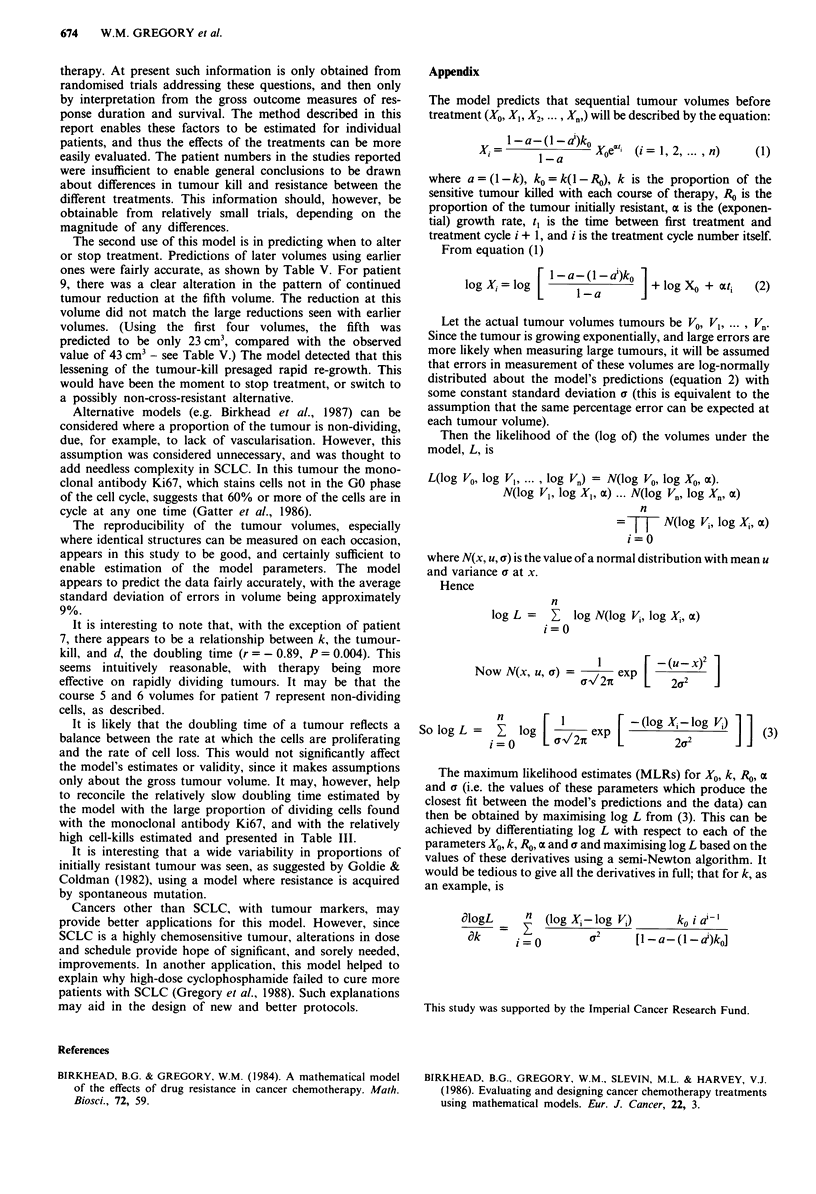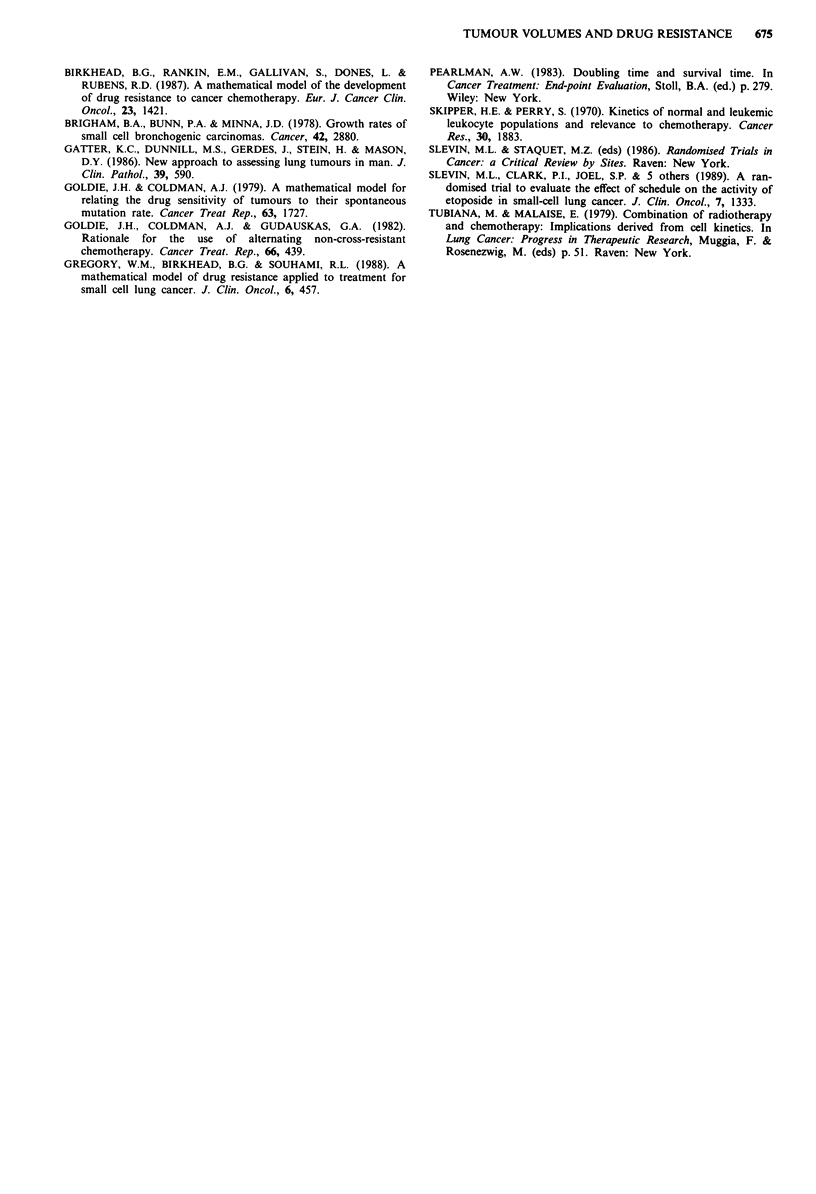# Using mathematical models to estimate drug resistance and treatment efficacy via CT scan measurements of tumour volume.

**DOI:** 10.1038/bjc.1990.354

**Published:** 1990-10

**Authors:** W. M. Gregory, R. H. Reznek, M. Hallett, M. L. Slevin

**Affiliations:** Medical Oncology Unit, Guy's Hospital, London, UK.

## Abstract

A previously described mathematical model designed to evaluate resistance and tumour-kill for individual patients, and to predict changing tumour sizes, has been applied to patients with small cell lung cancer. The model requires tumour volume measurements, and these were obtained via computed tomography scans of the chest. The model fitted the data well, and was able to predict later tumour volumes using earlier ones, as well as suggesting times at which to change or abandon treatment for individual patients. The model gave estimates for resistance and tumour-kill which may provide additional useful outcome measures for clinical trials, and help in the design of future studies.


*Br. J. Cancer* (1990), **62**, 671–675 © Macmillan Press Ltd., 1990

### Using mathematical models to estimate drug resistance and treatment efficacy via CT scan measurements of tumour volume

W.M. Gregory[1], R.H. Reznek[2], M. Hallett[2] & M.L. Slevin[2]

[1]*Medical Oncology Unit, Guy's Hospital, St Thomas's Street, London SE1 9RT; and* [2]*Departments of Radiology and Medical Oncology, St Bartholomew's Hospital, London EC1A 7BE, UK.*

**Summary** A previously described mathematical model designed to evaluate resistance and tumour-kill for individual patients, and to predict changing tumour sizes, has been applied to patients with small cell lung cancer. The model requires tumour volume measurements, and these were obtained via computed tomography scans of the chest. The model fitted the data well, and was able to predict later tumour volumes using earlier ones, as well as suggesting times at which to change or abandon treatment for individual patients. The model gave estimates for resistance and tumour-kill which may provide additional useful outcome measures for clinical trials, and help in the design of future studies.

The standard measures of success in clinical trials, namely differences in response rates, response duration or survival, although essential, yield little information as to why a particular regime fares better, or by what means prognosis is improved. Consequently the rationale for proceeding from one trial to the next is often unclear, many trials are needed to establish the principles on which development may take place, and trials often seem to produce contradictory results (Slevin & Staquet, 1986).

Attempts have been made, in recent years, to explain and interpret results from clinical trials in terms of resistance to and efficacy of chemotherapy, and differences in tumour growth rates using mathematical models (Birkhead & Gregory, 1984; Birkhead *et al.*, 1986; Goldie & Coldman, 1979; Skipper & Perry 1970). Such models, it is hoped, will provide meaningful explanations for trial differences, improving the speed and direction of research.

The models of Goldie & Coldman (1979) and Skipper & Perry (1970) espouse general principles, such as the alternating of non-cross-resistant drug combinations. A model has also been developed for individual patients (Birkhead & Gregory, 1984; Birkhead *et al.*, 1986). It was thought that, once validated, such a model might enable results to be achieved on smaller numbers of patients, since the additional interpretative information ought to improve the power of any tests used. An attempt is made to validate this model on patients with small cell lung cancer (SCLC). The model requires an accurate method of measuring tumour volumes, and computerised tomography (CT) scans of the chest have been employed to this end. Having estimated the resistance to and efficacy of chemotherapy, and the tumour growth rate, the model predicts the sequence of tumour volumes before each course of chemotherapy. The validity and accuracy of these predictions were tested on a series of up to seven scans on each of nine patients with SCLC.

### The model

The model seeks to relate changing tumour volumes to proportions of sensitive and resistant tumour, and to tumour growth rate. This is represented diagrammatically in Figure 1. Resistant tumour is assumed to be tumour which can never be killed with the given drug dose due to inherent (cellular) resistance. The remaining tumour is considered sensitive, although not all of it will be killed by a single administration of the drug, due to such factors as cells not being in cycle, uneven drug distribution, problems of blood

**Figure 1** Diagrammatic representation of the model.

supply and the likely stochastic nature of cell-killing by cytotoxic agents. The tumour growth rate is empirically assumed to be exponential for the period of therapy, and it is assumed that throughout the treatment period the mutation rate from sensitivity to resistance or vice versa is negligible in comparison to the other effects.

The proportion of sensitive tumour killed by each cycle of the treatment is assumed to be the same (Skipper & Perry, 1970), and is represented by $k$. The proportion of tumour initially resistant is represented by $R_0$. The tumour doubling time is denoted by $d$. The model predicts, for particular values of these three independent variables, $k$, $R_0$ and $d$, given the above assumptions, the sequence of tumour volumes before each treatment cycle (Birkhead & Gregory, 1984), as shown in the Appendix.

### Patients and methods

### *Patients*

Nine patients with SCLC had tumour volumes measured. They were taken from two separate trials, one comparing etoposide and adriamycin (VA) with oncovin, etoposide and adriamycin (OVA) in limited disease patients, the other comparing two different schedules of etoposide given as a single agent in extensive disease (Slevin *et al.*, 1989), the same dose of etoposide being given as a continuous infusion for 1 day, or as separate 2 h continuous infusions over 5 days.

The observed tumour volumes along with the times (in days) since the start of treatment, at which the scans were

Correspondence: W.M. Gregory.
Received 25 July 1989; and in revised form 10 May 1990.

taken, are given in Table I, and shown diagrammatically in Figures 2 and 3. Patients with peripheral masses on chest X-ray were chosen for the study since it was possible to separate tumour from mediastinal structures on the scans in these patients. The patients were scanned on a GE 9800 Whole Body Scanner. Scans were performed at 1 cm intervals throughout the region of the tumour. Where necessary, a bolus of intravenous contrast medium was administered to delineate vascular structures. The area of the lesion was then calculated on each image using a tracing device. As the scan thickness was 1 cm in each image the volume could be easily estimated. Care was taken to avoid measuring areas of lung consolidation or collapse, although this was not always possible. Where such discrimination was difficult in a series of scans a special effort was made to measure the same structures on each scan in the series. However, the initial measurements in this series were often made as the scans became available, several weeks apart.

One patient died during therapy and consequently has only three tumour volumes recorded; the rest have at least four, generally five, and in one case seven tumour volumes measured.

### *Methods*

In order to estimate the model's parameters, i.e. the proportion of sensitive tumour killed with each cycle of therapy, $k$, the resistance at presentation $R_0$, and the tumour doubling time $d$, four tumour volumes are required. Hence the model could only be applied to eight of the nine patients.

With only four volumes, if the model fits the observed data

**Table I** Tumour volumes ($cm^3$) and times (in days since start of treatment) at which scans were performed

| | Pre-course | | | | | | |
|---|---|---|---|---|---|---|---|
| *Patient* | *1* | *2* | *3* | *4* | *5* | *6* | *7* |
| 1 Volume | 15.2 | 7.65 | 4.96 | 3.84 | 2.41 | 2.53 | 1.66 |
| Time | 0 | 23 | 44 | 65 | 86 | 107 | 135 |
| 2 Volume | 46.7 | 29.7 | 27.1 | 23.0 | 23.2 | | |
| Time | 0 | 20 | 50 | 78 | 103 | | |
| 3 Volume | 231.7 | 176.8 | 100.9 | 111.0 | | | |
| Time | 0 | 25 | 46 | 68 | | | |
| 4 Volume | 84.2 | 56.3 | 37.3 | 30.9 | | | |
| Time | 0 | 24 | 39 | 71 | | | |
| 5 Volume | 12.94 | 4.66 | 1.43 | 0.56 | 0.49 | | |
| Time | 0 | 28 | 42 | 68 | 88 | | |
| 6 Volume | 27.1 | 13.3 | 12.4 | | | | |
| Time | 0 | 31 | 55 | | | | |
| 7 Volume | 98.45 | 4.1 | 1.0 | ? | 0.79 | 0.80 | |
| Time | 0 | 24 | 42 | 68 | 94 | 115 | |
| 8 Volume | 111.7 | 70.6 | 24.0 | 17.5 | 9.7 | | |
| Time | 0 | 21 | 42 | 63 | 84 | | |
| 9 Volume | 745.0 | 380.5 | 197.2 | 52.6 | 43.4 | 120.8 | |
| Time | 0 | 27 | 48 | 72 | 91 | 118 | |

? = scan not done.

**Figure 2** Comparison of observed tumour volumes (continuous lines) with those predicted by the model (dashed lines) for patients 1–5.

at all, it will fit exactly, since all four volumes will be needed to estimate the parameters (given four tumour volumes, equation (1) in the Appendix will generate three equalities, just sufficient to derive values for the three independent parameters, $k$, $R_0$ and $d$). If the model is not a reasonable representation of the actual diseases processes, it may be expected that no values of the parameters would be capable of predicting the observed volumes. For instance, if the percentage tumour reduction on the first cycle of treatment was less than that seen on the second cycle (assuming a similar interval between cycles) the model would be invalid. With more than four tumour volumes the accuracy of the model can be evaluated, assuming the model fits at all, as just explained, since its consistency in predicting the sequential tumour volumes can be examined. In these cases (six of the nine patients), all the tumour volumes were used to estimate the model's parameters. The model can then be validated by a $\chi^2$ test comparing the observed tumour volumes with those expected under the model assumptions.

Supposing $k$, $R_0$ and $d$ were known, some slight differences would still be expected between the model predictions and the actual tumour volumes, due to inaccuracies in measurement, i.e. the variations in marking out the area of tumour or delineating the tumour from other structures, as well as collapse and consolidation within the tumour. Hence in order to estimte $k$, $R_0$ and $d$, a normal distribution of errors about these predictions has been assumed, the mathematics of which is given in the Appendix. The variance of this distribution will reflect differences between the observed tumour volumes and the model's predictions, and will thus measure the accuracy of the model. Furthermore, in the patients with more than four volumes, since $k$, $R_0$ and $d$ can be estimated from just four volumes, these estimates can be used to predict the remaining volumes, providing a further substantive test of the model's validity. A computer program has been written, in Microsoft FORTRAN 77 for IBM compatible microcomputers, to produce the estimates, and is available on request. The estimation procedure takes only a few seconds to run.

### Results

### *Reproducibility of volume estimates*

To test the reproducibility and accuracy of the CT volume estimates, four of the nine patients' volumes were independently re-measured. The pairs of volumes for these four patients are given in Table II. Considerable variability was found in these estimates, with the mean error being 17%. It appeared that in some cases adjacent normal structures were included in the measurement on one occasion but not on the other. When exactly the same structures were included in the measurements, the results were consistent, and the measurements were in close agreement.

### *Model estimates*

The estimates of sensitive tumour kill, resistance and tumour volume doubling time for each of the eight patients are

**Table II** Pairs of repeated tumour volume measurements

| | Volumes before course ($cm^3$) | | | | | |
|---|---|---|---|---|---|---|
| *Patient* | *1* | *2* | *3* | *4* | *5* | *6* |
| 3 | 231.70 | 176.80 | 100.90 | 111.00 | | |
| | 230.70 | 143.70 | 100.80 | 110.50 | | |
| 7 | | | 1.00 | | 0.79 | 0.80 |
| | | | 1.35 | | 0.96 | 0.61 |
| 8 | 111.70 | 70.60 | 24.00 | 17.50 | 9.70 | |
| | 93.19 | 61.01 | 28.03 | 24.27 | 16.04 | |
| 9 | 745.00 | 380.50 | 197.20 | 52.60 | 43.36 | 120.80 |
| | 711.67 | 432.22 | 176.60 | 55.20 | 39.00 | 166.00 |

shown in Table III. A detailed worked example showing how the estimates were derived for patient 9 is shown in Table IV. Initially, a guess is made for the values of the parameters (see Table IV). The model's predictions, based on these guesses, are then compared with the actual results (by evaluating the log-likelihood as described in the Appendix). A new estimate of the four parameters is produced based on the differences between the predictions and actuals (this involves using the semi-Newton alguritmm described in the Appendix). This new estimate should be closer to the actuals (and thus have a greater likelihood). This procedure is repeated until the predictions come no closer to the actuals (i.e. the likelihood no longer increases significantly). The likelihood for each of the volumes, given the final 'best' parameters, is given in Table IV, along with a comparison of the predictions with the actual volumes.

In three patients the estimates for tumour volume doubling time were very long, implying a very slow growth rate. In such cases, with the tumour growing so slowly, very small volume changes would need to be detectable in order to estimate the doubling time over the short time intervals considered. Inaccuracies in the volumes measurements themselves, as previously calculated, are at least as great as these changes, making estimates of the doubling time unreliable in such cases. The doubling time in these patients has thus been assumed to be approximately 150 days, based on the estimates of others for the extremes in doubling time in SCLC (Brigham *et al.*, 1978; Tubiana & Malaise, 1979; Pearlman, 1983). This problem does not significantly affect the estimates for resistance and tumour-kill, which are less sensitive to small volume differences.

The accuracy of the model's predictions (see Appendix), measured by standard deviations of errors about the model's predictions (given as percentages of the tumour volumes), are also shown in Table III. The mean percentage standard deviation of these errors in prediction was 6.5%, excluding the patients with only four volumes measured, where the predictions matched the observed volumes. This percentage error is within the likely errors resulting from inaccuracies in the measurements, as described previously, and confirms that the model provides a good fit to the data. This can be seen in Figures 2 and 3, which plot the observed volumes against the predictions. The $\chi^2$ goodness-of-fit tests supported this finding.

The tumour volumes for the patients with more than four volumes were used to investigate the consistency of the model's predictions, and to see whether the model could be used in a predictive sense, for instance in deciding when to change or to abandon a particular treatment. In these patients, the first four volumes alone were used to estimate the sensitive tumour-kill, resistance and doubling time. These estimates were then used to try and predict the later volumes. These predictions, along with the actual, measured volumes, are given in Table V. For patient 9, the predicted volumes for courses 5 and 6, using the first four volumes, bore no resemblance to the actual volumes. A further prediction of the course 6 volume, using the first five volumes, was also made for this patient, and this prediction is included in Table V. For the other patients the predictions are close to the actual volumes.

### Discussion

This mathematical model has two important potential uses. First, it may provide an important short-cut to obtaining information about resistance to and efficacy of chemo-

**Table III** Estimates of sensitive tumour-kill ($k$), resistance ($R_o$) and doubling time ($d$) for the nine patients

| *Patient* | *Number of scans* | *Regime*[a] | *k (%)* | $R_o$ *(%)* | *d (days)* | *Likely % error*[b] |
|---|---|---|---|---|---|---|
| 1 | 7 | OVA | 46 | 11 | >150 | 11 |
| 2 | 5 | VP5 | 66 | 0.85 | 23 | 2 |
| 3 | 4 | VP1 | 92 | 0.06 | 8 | 0 |
| 4 | 4 | VP1 | 49 | 9 | 88 | 0 |
| 5 | 5 | VP5 | 81 | 0.36 | 30 | 7 |
| 6 | 3 | VP1 | ? | ? | ? | |
| 7 | 5 | VA | 97 | 0.84 | >150 | 4 |
| 8 | 5 | OVA | 59 | 2 | 92 | 15 |
| 9 | 6 | VP5 | 90 | 0.01 | 12 | 19 |

[a]OVA: oncovin, vincristine, adriamycin. VA: vincristine, adriamycin. VP1: VP16 given over 1 day. VP5: VP16 given over 5 days. [b]One standard deviation of errors about model predictions (see Appendix). ? = insufficient scans to apply model.

**Table IV** A worked example for patient 9, including a comparison of model predictions with actual volumes

Initial guesses for the parameters were:
$k = 0.8$, $R_0 = 0.0005$, $d = 14$ days, s.d. ($\sigma$) = 0.2, $X_0 = 745$ cm$^3$.
(The initial log-likelihood was −48.263).

The semi-Newton maximisation routine produced the 'best' (or maximum likelihood) estimates at the following parameter values:
$k = 0.90$, $R_0 = 0.0001$, $d = 11.7$ days, s.d. ($\sigma$) = 0.169, $X_0 = 793$ cm$^3$.
(The maximum log-likelihood was 2.163).

The actual values and predictions were as follows

| *Course*[a] *(i)* | *Time (days)* | *Actuals* $X_i$ | *Actuals* *Log* $(X_i)$ | *Predictions* *u* | *Predictions* *Log (u)* | *Likelihood* *L* | *Likelihood* *Log (L)* |
|---|---|---|---|---|---|---|---|
| 0 | 0 | 745.00 | 6.61 | 793.07 | 6.68 | 2.21 | 0.79 |
| 1 | 27 | 380.50 | 5.94 | 400.91 | 5.99 | 2.25 | 0.81 |
| 2 | 48 | 197.25 | 5.28 | 143.62 | 4.97 | 0.40 | −0.91 |
| 3 | 72 | 52.60 | 3.96 | 67.36 | 4.21 | 0.81 | −0.21 |
| 4 | 91 | 43.60 | 3.78 | 41.54 | 3.73 | 2.27 | 0.82 |
| 5 | 118 | 120.80 | 4.79 | 121.21 | 4.80 | 2.36 | 0.86 |

[a]Course 0 is the pre-treatment value.

**Figure 3** Comparison of observed tumour volumes (continuous lines) with those predicted by the model (dashed lines) for patients 6–9.

**Table V** Model predictions of later tumour volumes from earlier tumour volumes

| *Patient* | *Number of volumes used in prediction* | *Actuals/ predictions* | *Course 5* | *Course 6* | *Course 7* |
|---|---|---|---|---|---|
| 1 | 4 | actuals | 2.41 | 2.53 | 1.66 |
| | | predictions | 2.50 | 2.11 | 1.88 |
| 2 | 4 | actuals | 23.2 | | |
| | | predictions | 17.6 | | |
| 5 | 4 | actuals | 0.49 | | |
| | | predictions | 0.26 | | |
| 7 | 4 | actual | | 0.80 | |
| | | predictions | | 0.83 | |
| 8 | 4 | actual | 9.7 | | |
| | | predictions | 11.3 | | |
| 9 | 4 | actual | 43.4 | 120.8 | |
| | | predictions | 23.0 | 11.0 | |
| 9 | 5 | actual | | 120.8 | |
| | | predictions | | 174.3 | |

therapy. At present such information is only obtained from randomised trials addressing these questions, and then only by interpretation from the gross outcome measures of response duration and survival. The method described in this report enables these factors to be estimated for individual patients, and thus the effects of the treatments can be more easily evaluated. The patient numbers in the studies reported were insufficient to enable general conclusions to be drawn about differences in tumour kill and resistance between the different treatments. This information should, however, be obtainable from relatively small trials, depending on the magnitude of any differences.

The second use of this model is in predicting when to alter or stop treatment. Predictions of later volumes using earlier ones were fairly accurate, as shown by Table V. For patient 9, there was a clear alteration in the pattern of continued tumour reduction at the fifth volume. The reduction at this volume did not match the large reductions seen with earlier volumes. (Using the first four volumes, the fifth was predicted to be only 23 $cm^3$, compared with the observed value of 43 $cm^3$ - see Table V.) The model detected that this lessening of the tumour-kill presaged rapid re-growth. This would have been the moment to stop treatment, or switch to a possibly non-cross-resistant alternative.

Alternative models (e.g. Birkhead *et al.*, 1987) can be considered where a proportion of the tumour is non-dividing, due, for example, to lack of vascularisation. However, this assumption was considered unnecessary, and was thought to add needless complexity in SCLC. In this tumour the monoclonal antibody Ki67, which stains cells not in the G0 phase of the cell cycle, suggests that 60% or more of the cells are in cycle at any one time (Gatter *et al.*, 1986).

The reproducibility of the tumour volumes, especially where identical structures can be measured on each occasion, appears in this study to be good, and certainly sufficient to enable estimation of the model parameters. The model appears to predict the data fairly accurately, with the average standard deviation of errors in volume being approximately 9%.

It is interesting to note that, with the exception of patient 7, there appears to be a relationship between $k$, the tumour-kill, and $d$, the doubling time ($r = -0.89$, $P = 0.004$). This seems intuitively reasonable, with therapy being more effective on rapidly dividing tumours. It may be that the course 5 and 6 volumes for patient 7 represent non-dividing cells, as described.

It is likely that the doubling time of a tumour reflects a balance between the rate at which the cells are proliferating and the rate of cell loss. This would not significantly affect the model's estimates or validity, since it makes assumptions only about the gross tumour volume. It may, however, help to reconcile the relatively slow doubling time estimated by the model with the large proportion of dividing cells found with the monoclonal antibody Ki67, and with the relatively high cell-kills estimated and presented in Table III.

It is interesting that a wide variability in proportions of initially resistant tumour was seen, as suggested by Goldie & Coldman (1982), using a model where resistance is acquired by spontaneous mutation.

Cancers other than SCLC, with tumour markers, may provide better applications for this model. However, since SCLC is a highly chemosensitive tumour, alterations in dose and schedule provide hope of significant, and sorely needed, improvements. In another application, this model helped to explain why high-dose cyclophosphamide failed to cure more patients with SCLC (Gregory *et al.*, 1988). Such explanations may aid in the design of new and better protocols.

### Appendix

The model predicts that sequential tumour volumes before treatment ($X_0, X_1, X_2, \ldots, X_n$) will be described by the equation:

$$X_i = \frac{1-a-(1-a^i)k_0}{1-a} X_0 e^{\alpha t_i} \quad (i = 1, 2, \ldots, n) \qquad (1)$$

where $a = (1-k)$, $k_0 = k(1-R_0)$, $k$ is the proportion of the sensitive tumour killed with each course of therapy, $R_0$ is the proportion of the tumour initially resistant, $\alpha$ is the (exponential) growth rate, $t_i$ is the time between first treatment and treatment cycle $i+1$, and $i$ is the treatment cycle number itself.

From equation (1)

$$\log X_i = \log \left[ \frac{1-a-(1-a^i)k_0}{1-a} \right] + \log X_0 + \alpha t_i \qquad (2)$$

Let the actual tumour volumes tumours be $V_0, V_1, \ldots, V_n$. Since the tumour is growing exponentially, and large errors are more likely when measuring large tumours, it will be assumed that errors in measurement of these volumes are log-normally distributed about the model's predictions (equation 2) with some constant standard deviation $\sigma$ (this is equivalent to the assumption that the same percentage error can be expected at each tumour volume).

Then the likelihood of the (log of) the volumes under the model, $L$, is

$$L(\log V_0, \log V_1, \ldots, \log V_n) = N(\log V_0, \log X_0, \alpha). N(\log V_1, \log X_1, \alpha) \ldots N(\log V_n, \log X_n, \alpha) = \prod_{i=0}^{n} N(\log V_i, \log X_i, \alpha)$$

where $N(x, u, \sigma)$ is the value of a normal distribution with mean $u$ and variance $\sigma$ at $x$.

Hence

$$\log L = \sum_{i=0}^{n} \log N(\log V_i, \log X_i, \alpha)$$

$$\text{Now } N(x, u, \sigma) = \frac{1}{\sigma\sqrt{2\pi}} \exp \left[ \frac{-(u-x)^2}{2\sigma^2} \right]$$

$$\text{So } \log L = \sum_{i=0}^{n} \log \left[ \frac{1}{\sigma\sqrt{2\pi}} \exp \left[ \frac{-(\log X_i - \log V_i)}{2\sigma^2} \right] \right] \qquad (3)$$

The maximum likelihood estimates (MLRs) for $X_0$, $k$, $R_0$, $\alpha$ and $\sigma$ (i.e. the values of these parameters which produce the closest fit between the model's predictions and the data) can then be obtained by maximising $\log L$ from (3). This can be achieved by differentiating $\log L$ with respect to each of the parameters $X_0$, $k$, $R_0$, $\alpha$ and $\sigma$ and maximising $\log L$ based on the values of these derivatives using a semi-Newton algorithm. It would be tedious to give all the derivatives in full; that for $k$, as an example, is

$$\frac{\partial \log L}{\partial k} = \sum_{i=0}^{n} \frac{(\log X_i - \log V_i)}{\sigma^2} \frac{k_0\, i\, a^{i-1}}{[1-a-(1-a^i)k_0]}$$

This study was supported by the Imperial Cancer Research Fund.

### References

BIRKHEAD, B.G. & GREGORY, W.M. (1984). A mathematical model of the effects of drug resistance in cancer chemotherapy. *Math. Biosci.*, **72**, 59.

BIRKHEAD, B.G., GREGORY, W.M., SLEVIN, M.L. & HARVEY, V.J. (1986). Evaluating and designing cancer chemotherapy treatments using mathematical models. *Eur. J. Cancer*, **22**, 3.

BIRKHEAD, B.G., RANKIN, E.M., GALLIVAN, S., DONES, L. & RUBENS, R.D. (1987). A mathematical model of the development of drug resistance to cancer chemotherapy. *Eur. J. Cancer Clin. Oncol.*, **23**, 1421.

BRIGHAM, B.A., BUNN, P.A. & MINNA, J.D. (1978). Growth rates of small cell bronchogenic carcinomas. *Cancer*, **42**, 2880.

GATTER, K.C., DUNNILL, M.S., GERDES, J., STEIN, H. & MASON, D.Y. (1986). New approach to assessing lung tumours in man. *J. Clin. Pathol.*, **39**, 590.

GOLDIE, J.H. & COLDMAN, A.J. (1979). A mathematical model for relating the drug sensitivity of tumours to their spontaneous mutation rate. *Cancer Treat Rep.*, **63**, 1727.

GOLDIE, J.H., COLDMAN, A.J. & GUDAUSKAS, G.A. (1982). Rationale for the use of alternating non-cross-resistant chemotherapy. *Cancer Treat. Rep.*, **66**, 439.

GREGORY, W.M., BIRKHEAD, B.G. & SOUHAMI, R.L. (1988). A mathematical model of drug resistance applied to treatment for small cell lung cancer. *J. Clin. Oncol.*, **6**, 457.

PEARLMAN, A.W. (1983). Doubling time and survival time. In *Cancer Treatment: End-point Evaluation*, Stoll, B.A. (ed.) p. 279. Wiley: New York.

SKIPPER, H.E. & PERRY, S. (1970). Kinetics of normal and leukemic leukocyte populations and relevance to chemotherapy. *Cancer Res.*, **30**, 1883.

SLEVIN, M.L. & STAQUET, M.Z. (eds) (1986). *Randomised Trials in Cancer: a Critical Review by Sites*. Raven: New York.

SLEVIN, M.L., CLARK, P.I., JOEL, S.P. & 5 others (1989). A randomised trial to evaluate the effect of schedule on the activity of etoposide in small-cell lung cancer. *J. Clin. Oncol.*, **7**, 1333.

TUBIANA, M. & MALAISE, E. (1979). Combination of radiotherapy and chemotherapy: Implications derived from cell kinetics. In *Lung Cancer: Progress in Therapeutic Research*, Muggia, F. & Rosenezwig, M. (eds) p. 51. Raven: New York.